# Simulated Gastrointestinal Digestion of Bioprocessed Spelt Seeds: Bioaccessibility and Bioactivity of Phenolics

**DOI:** 10.3390/antiox11091703

**Published:** 2022-08-30

**Authors:** Marjeta Mencin, Maja Mikulič Petkovšek, Robert Veberič, Petra Terpinc

**Affiliations:** 1 Department of Food Science and Technology, Biotechnical Faculty, University of Ljubljana, Jamnikarjeva 101, SI-1111 Ljubljana, Slovenia; 2 Department of Agronomy, Biotechnical Faculty, University of Ljubljana, Jamnikarjeva 101, SI-1111 Ljubljana, Slovenia

**Keywords:** spelt seeds, bioaccessibility, biostability, bioprocessing, in vitro digestion, extractable and bound phenolics

## Abstract

The goal of this research was to evaluate the impact of different bioprocessing techniques on improved bioaccessibility of phenolics from spelt seeds. Despite the negative influence of gastrointestinal digestion, fermentation of germinated seeds significantly increased the bioaccessibility of total phenolics and their antioxidant activity compared to digested raw seeds. Enzymatic treated fermented seeds showed the highest relative bioaccessibility of *p*-coumaric and *trans*-ferulic acids, while their absolute contents were significantly higher in “germinated + fermented” seeds. Our research suggests that pretreatment of spelt seeds with hydrolytic enzymes improves access of fermenting microorganisms to structural elements, resulting in an increased content of extractable and bound *trans*-ferulic acid. Significantly higher biostability of phenolics was observed in raw seeds. Some major quality changes in the composition of extracts were observed under simulated in vitro digestion, since antioxidants of the same extract showed a different relative decrease in DPPH^•^ and ABTS^•+^ scavenging activities compared to the raw seeds or their corresponding undigested counterparts. It is therefore important to increase the content of extractable antioxidants in seeds by bioprocessing, since they are strongly diminished during digestion.

## 1. Introduction

Foods rich in antioxidants have attracted attention, since their consumption plays an essential role in the prevention of various diseases. In order to exert a health-promoting effect, phytochemicals must first survive food processing, be successfully released from the food matrix, be able to pass through the intestinal epithelia, and express (as the parent compound or its metabolite) bioactive properties in the target tissue [1]. Phenolics in cereals can prevent oxidation and reactive oxygen species (ROS) formation, but their antioxidant properties depend not only on their structure and concentrations in cereals but also on their bioaccessibility [2]. Bioaccessibility refers to the amount of compound released from the solid food matrix into the intestine during gastrointestinal (GI) digestion [3]. Approximately 99% of spelt major phenolics, *trans*-ferulic and *p*-coumaric acids, are present in an insoluble form and are bound to indigestible cell wall components [4]; their structural position within these building blocks may therefore hinder the access of feruloyl esterase, cellulase, and xylanase, enzymes that can release bound phenolics [5]. Soluble phenolics, which are located in vacuole, are generally more likely to be absorbed into systemic circulation, while unabsorbed phenolics are transported to the colon, where they can be biotransformed by microbial fermentation [6]. 

Bioprocessing techniques (germination/fermentation/enzymatic treatment) have been shown to enhance the bioaccessibility of phenolics from cereal bran incorporated into food matrices prior to consumption. Koistinen et al. [7] investigated the impacts of yeast fermentation and enzymatic treatment on the bioaccessibility of phenolics from wheat bread enriched with bioprocessed rye bran using an in vitro colon model. They showed that phenolic acids were released more extensively in bioprocessed bread, which was confirmed by a significant decrease in bound phenolic acids in enriched bread compared to non-enriched bread, following enzymatic digestion and dialysis. Furthermore, Anson et al. [2] showed that a combination of external enzymes and fermentation was the most effective treatment that increased the bioaccessibility of ferulic acid from 1.1% to 5.5%.

The in vitro GI digestion model is an effective tool for evaluating the bioaccessibility of bioactive compounds, because it simulates the oral, gastric and small intestinal phases and, occasionally, fermentation in the colon [8]. Indeed, it is relatively inexpensive, rapid, not subject to ethical constraints, conditions can be controlled, sampling is simple and results are reproducible. In vitro digestion models have already been used to mimic the physiological release of phenolics in some cereals and cereal-based products [9,10]. However, the release of phenolics from bioprocessed *Triticum* matrix under digestion has not yet been reported. The objectives of the present study were therefore (a) to determine the content of total and individual phenolics and their antioxidant activity after homogenised spelt seeds that had been subjected to in vitro GI digestion (bioaccessible phenolics), and (b) to evaluate the percentage of bioaccessibility of these same phenolics after the seeds had been treated with different bioprocessing techniques and their combinations (germination/fermentation/enzymatic treatment).

## 2. Materials and Methods

### 2.1. Chemicals

Methanol (99.9%), formic acid, sodium hydroxide, sodium dihydrogen phosphate dehydrate, sodium carbonate and calcium chloride dehydrate (CaCl_2_(H_2_O)_2_) were purchased from Merck (Darmstadt, Germany). Folin–Ciocalteu reagent, 2,2-diphenyl-1-picrylhydrazyl radical (DPPH•) reagent, 2,2-Azino-bis-3-etilbenzotiazolin-6-sulfonic acid (ABTS) reagent, Trolox, hydrochloric acid, *p*-coumaric acid, *trans*-ferulic acid, caffeic acid, *p*-hydroxybenzoic acid, gallocatechin, 4-vinylphenol, 4-vinylguaiacol, α-amylase (EC 232-565-6) from porcine pancreas (enzyme activity 5 U/mg solid), pepsin (EC 232-629-3) from porcine gastric mucosa (enzyme activity ≥ 2500 U/mg protein) and pancreatin (EC 232-468-9) from porcine pancreas (4 × USP specifications) were purchased from Sigma-Aldrich (Steinheim, Germany). Bile bovine (EC 232-369-0) was from Millipore (ZDA). Potassium chloride (KCl), potassium dihydrogen phosphate (KH_2_PO_4_), sodium bicarbonate (NaHCO_3_), sodium chloride (NaCl), and magnesium chloride hexahydrate (MgCl_2_(H_2_O)_6_) were purchased from Sigma-Aldrich (Steinheim, Germany). Ammonium carbonate ((NH_4_)_2_CO_3_) was from Honeywell/Riedel-de Haen (Seelze, Germany). All chemicals and reagents used were of analytical quality. For preparation of working solutions ultrapure water (Milli-Q; Millipore, Bedford, MA, USA) was used.

### 2.2. Sample Preparation

Spelt (*Triticum spelta* L. cv. Ostro) seeds were obtained from Slovenia. Seeds were germinated according to Mencin et al. [4] under specific abiotic stress (darkness, at 25 °C for 144 h, with the addition of 25 mM NaCl after 48 h and 50 mM sorbitol after 96 h of germination) and then immediately milled (particles size < 0.25 mm), freeze-dried, and stored at –20 °C. The milled and freeze-dried raw spelt seeds were fermented according to [11], carried out for 72 h at 30 °C under static conditions, a sample-to-saline ratio 1:1.5 (10 g:15 mL), and with the addition of 0.75 mL of *Saccharomyces cerevisiae* inoculum. Enzymatic treatment of milled and freeze-dried raw spelt seeds was carried out at 40 °C for 4 h, with the addition of cellulase (C) (25 U/g DW), xylanase (X) (5 U/g DW), feruloyl esterase (E) (10 U/g DW), protease (P) (50 U/g DW), and α-amylase (A) (50 U/g DW). Other samples we used for in vitro GI digestion were: germinated spelt seeds treated with a combination of all five enzymes (C+X+E+A+P) (hereinafter referred to as “germinated + enzymatic treated”); fermented seeds with *S. cerevisiae* treated with C+X+E+A+P (hereinafter referred to as “fermented + enzymatic treated”), germinated spelt seeds fermented with *S. cerevisiae* (hereinafter referred to as “germinated + fermented”), and enzymatic treated seeds (C+X+E+A+P) fermented with *L. plantarum* (hereinafter referred to as “enzymatic treated + fermented”). Prior to analysis, raw and bioprocessed spelt seeds were freeze-dried (–50 °C and 30 mTorr) to a moisture content < 5%. We selected optimal treatments of spelt seeds based on our previous studies on the germination, fermentation, and enzymatic treatment of spelt seeds. 

### 2.3. Simulated In Vitro Gastrointestinal Digestion of Spelt Seeds

In vitro GI digestion consisted of three-steps (oral, gastric and intestinal digestion) and was adapted from Brodkorb et al. [12] and Minekus et al. [13], with slight modifications. The solutions used to simulate GI digestive fluids (SSF: simulated salivary fluid, SGF: simulated gastric fluid, and SIF: simulated intestinal fluid) were prepared according to Table 1. Briefly, in oral digestion, 1.67 g of sample (raw or bioprocessed spelt seeds) was placed into a 50 mL polyethylene centrifuge tube and mixed with 2.67 mL of SSF to create a thin paste-like consistency. We then added 31.3 mg of α-amylase (to achieve 75 U/mL in the final mixture), followed by 12.5 µL of 0.3 M calcium chloride dehydrate and 666 µL of water and thoroughly mixed. The solution was incubated in a Roto-Therm incubated rotator (Benchmark, Edison, NJ, USA) at 37 °C for 2 min at constant rotation combined with oscillations. Afterward, gastric digestion was continued by the immediate addition of 4 mL SGF, 8 mg of pepsin (to achieve 2000 U/mL in the final mixture) and 2.5 µL of 0.3 M calcium chloride dehydrate. The mixture was intensively mixed, the pH of the solution was adjusted to 3 with 1 M HCl, and the volume was brought to 10 mL with water. The mixture was incubated in a Roto-Therm incubated rotator at 37 °C for 2 h. In order to simulate intestinal digestion, 8 mL of SIF, 20 mg of pancreatin (to achieve 100 U/mL of trypsin activity in the final mixture), 80 mg of bile bovine (to achieve 10 mM in the final mixture), and 20 µL of 0.3 M calcium chloride dehydrate were then added. The mixture was afterwards intensively mixed, and the pH of the solution was adjusted to 7 with 1 M NaOH and the volume was brought to 20 mL with water. The mixture was incubated in a Roto-Therm incubated rotator at 37 °C for 2 h. 

At the end of GI digestion, samples were immediately placed in an ice bath for 10 min to deactivate enzymes and then centrifuged for 10 min at 9793.9× *g* and 10 °C. The aliquots of supernatants (5 mL) were collected, filtered (pore size, 0.45 µm) and stored at 4 °C. The remaining residue was freeze-dried and stored at –20 °C until further analysis.

A reference blank (without the added sample) was incubated under the same conditions and used to determine phenolics content and antioxidant activity for the correction of interference from the digestive enzymes and buffers. Under the same enzymatic and incubation conditions, proteins (enzymes) can interfere with the analysis of phenolics (especially with the Folin-Ciocalteu reagent). Our controls (undigested samples) were spelt seeds exposed to simulated digestive fluids without enzymes or bile. For each sample employed for the in vitro GI digestion, three replicates were used.

### 2.4. Extraction of Extractable and Bound Phenolics

Extractable and bound phenolics were extracted according to the extraction procedure described in Mencin et al. [4]. Briefly, 1 g of the homogenised and freeze-dried undigested and digested spelt seeds were mixed with 9 mL of absolute methanol. After shaking for 2 h at room temperature, the samples were centrifuged (9793.9× *g*, 10 min) and filtered (pore size, 0.45 µm). These filtered supernatants from the methanolic extraction contained the extractable phenolics. The solid residues after methanol extraction were treated by adding 20 mL of 2 M sodium hydroxide and shaking for 4 h at room temperature. After hydrolysis, the samples were acidified to pH 3 with concentrated formic acid. These filtered hydrolysates represented a solution of bound phenolics. 

The supernatants and hydrolysates were further used for solid-phase extraction (SPE) for isolation and concentration of the phenolics from the extracts. SPE was performed according to [14]. Briefly, 100 mg Strata-X RP cartridges (Phenomenex, Torrance, CA, USA) were first preconditioned with 3 mL of 99.9% methanol, and then with 3 mL of water. The samples containing the extractable (5 mL) or bound (8 mL) phenolics were then added to the SPE cartridges and allowed to penetrate the matrix. The cartridges were washed with 4 mL of water and vacuum dried for 2 min. The phenolics were then eluted with 2 mL of 70% aqueous methanol. These methanolic eluates represented the corresponding extractable and bound fractions of phenolics. Until analysis, all samples were stored at 2 °C. 

### 2.5. Determination of Total Phenolics Content

Total phenolics contents (TPCs) were determined using the Folin–Ciocalteu method according to the method described in our previous publication [4]. Appropriately diluted samples were mixed with water, Folin-Ciocalteu reagent, and after 5 min with 20% sodium carbonate. The mixtures were allowed to incubate for 1 h at room temperature in the dark. Absorbance was read at 765 nm using a UV-visible spectrophotometer (model 8453; Hewlett Packard, Waldbronn, Germany). A standard curve was prepared with Trolox, and the final results are expressed as mg Trolox equivalents per g dry weight of freeze-dried spelt seeds (mg TE/g DW). 

### 2.6. Phenolics Profile

Analysis of individual phenolics was performed on HPLC (Thermo Dionex system; Thermo Scientific, San Jose, CA, USA), using a C18 column (Gemini C18; 150 mm × 4.6 mm; 3 µm; Phenomenex, Torrance, CA, USA) and an UV detector set at 280 nm and 310 nm. All phenolics were identified by mass spectrometry (LTQ XL linear ion trap mass spectrometer; Thermo Fisher Scientific, San Jose, CA, USA) with electrospray ionisation operating in negative- ion mode. All mass spectrometry conditions were described in our previous publication [4]. Identification and quantification of phenolics were confirmed by comparisons of their UV-VIS spectra and MS spectra and retention times with external standards and by internal standards, followed by fragmentation, as fully described in our previous investigation [4]. Concentrations of *p*-coumaric, *trans*-ferulic, caffeic and *p*-hydroxybenzoic acids and gallocatechin were calculated from the peak areas of the samples and the corresponding standards. Concentrations were expressed as µg per g spelt seeds DW (µg/g DW). One peak was tentatively identified as the *cis*-isomer of ferulic acid and was quantified using the calibration curve of *trans*-ferulic acid. For the compounds lacking standards, quantification was carried out using similar compounds as standards. Apigenin hexoside pentoside (I, II, III) and unknown C-glycosyl derivative were quantified according to *p*-coumaric acid. 

### 2.7. Determination of Antioxidant Activity

#### 2.7.1. DPPH^•^ Scavenging Activity

The antioxidant activity of phenolics from undigested and digested spelt seeds was determined using the DPPH assay as described in our previous paper [4]. Samples were added to 0.2 mM DPPH^•^ solution in 99.9% methanol. After the incubation period (1 h, at room temperature in the dark), the decrease in absorbance of the mixtures was measured at 520 nm. The final results are expressed as mg TE/g DW. 

#### 2.7.2. ABTS^•+^ Scavenging Activity

The scavenging activities of spelt seeds phenolics were also determined using ABTS assay, according to the method described by Mencin et al. [4]. The working solution of ABTS^•+^ was mixed with phosphate buffer (pH 7.4), water and the samples. The mixture was then incubated for 1 h in the dark. The decrease in absorbance of the reaction mixtures was measured at 734 nm. The final results are expressed as mg TE/g DW. 

### 2.8. Bioaccessibility and Biostability of Phenolics after Digestion

After GI digestion, we obtained two fractions: a soluble-extractable fraction (bioaccessible) (supernatant of digestion) and an insoluble-bound fraction (biostable) (residue of digestion). The relative bioaccessibility represents the percentage of phenolics released after simulated GI digestion that could become available for absorption into systemic circulation. The relative biostability indicates the percentage of phenolics that remain in the digested residue and are not released into the digestive tract. For total and individual phenolics, the proportion released from the spelt seed matrix into the digestive fluids and the proportion insoluble during digestion were calculated as follows:

Relative bioaccessibility (%) = (Supernatant/Undigested) ∗ 100(1)

Relative biostability (%) = (Residue /Undigested) ∗ 100(2)

where Supernatant (extractable phenolics) is the content of total (TPC) or individual phenolics in the supernatant after GI digestion, Residue (bound phenolics) is the TPC or individual phenolics content in the residue after digestion, and Undigested is the total phenolic content (extractable + bound) in samples exposed to simulated digestive fluids without enzymes or bile. All values were corrected (due to interference from the digestive enzymes and buffers) with reference blank samples.

### 2.9. Statistical Analysis

All analyses were performed in three parallel runs with two separate extractions. One-way analysis of variance (ANOVA) was performed for each parameter, followed by a Duncan‘s post-hoc test to determine significantly different means (*p* < 0.05) using the SPSS programme, version 22 for Windows (IBM, Armonk, NY, USA). In addition, the Pearson’s correlation coefficients (*r*) were determined to express the strength between two continuous variables. Multivariate statistical analysis (principal component analysis; PCA) was performed using OriginPro 2015 (OriginLab, Northampton, MA, USA) to interpret the differences in the analysed samples.

## 3. Results and Discussion

### 3.1. Total Phenolic Content

Considering the limitations of the Folin-Ciocalteu assay, the data obtained should always be interpreted with great caution, especially in situations in which, in addition to the studied extract or compound, the system contains complex food matrices. Because the Folin-Ciocalteu reagent can be reduced non-specifically by ascorbates, reducing sugars, aromatic amines, organic acids, fatty acids, proteins, and small peptides [15], we used a reference blank (without the added sample) to correct interference from the digestive enzymes and buffers. However, the contribution of digestive fluids, enzymes and bile to TPC is usually overlooked in these types of studies.

The content of TPCs for samples exposed to simulated digestive fluids without enzymes and bile (undigested samples) and samples after in vitro GI digestion (digested samples) are presented in Table 2. The TPCs of undigested seeds showed a predominance of bound phenolics in raw and with a single bioprocessing technique treated seeds, whereas the concentrations of extractable and bound phenolics were quite similar when different bioprocessing techniques were combined. Bioprocessing techniques had already resulted in an increased proportion of extractable TPCs over the total spelt phenolics in our previous experiments [4,11], but the seeds were analysed in the absence of digestive fluids. 

As shown in Table 2, in vitro digestion had a different effect on the content of phenolics in the extractable (bioaccessible) and bound (non-bioaccessible) fractions. Among the digested samples, the lowest extractable TPC was determined in raw seeds and the lowest bound TPC in enzymatic treated seeds (alone or in combination), while the highest extractable and bound TPCs were determined for “germinated + fermented” seeds. The considerable increase in extractable TPCs in the bioprocessed spelt seeds (a max 7-fold increase was observed in “germinated + fermented” seeds) compared to raw seeds after GI digestion may be due to the hydrolysis of various spelt fibre polymers resulting from the activity of the cereal and microbial enzymes, which may lead to a structural degradation of bran cell walls. Among the digested samples, germinated samples alone or in combination with fermentation or enzymatic treatment had higher extractable and bound TPCs than other bioprocessed samples. Only germinated seeds showed a statistically significant increase in extractable TPCs (by 16%) after digestion compared to the corresponding undigested seeds. The increase in the amount of extractable phenolics in digested germinated seeds may be the result of digestive enzymes and bile salts acting on the modified spelt seed matrix and facilitating the release of bound phenolics into the digestive juice [16]. Furthermore, the transition from an acidic to an alkaline environment leads to deprotonation of hydroxyl moieties of aromatic rings, which may have contributed to the increased extractable TPC of digested germinated seeds [17]. A possible reason may also be the structure of germinated seeds, since starch in germinated seeds is generally more digestible due to the enzymatically modified structure of starch granules, thin cell walls and readily available mono- and disaccharides. Polysaccharides in the cell walls are hydrolyzed during germination by de novo synthesized enzymes, resulting in changes in the composition of insoluble and soluble dietary fibre of cereal seeds [18]. Analogously, bound TPCs significantly decreased in almost all digested samples compared to the undigested ones, whereby the highest decrease (33%) was observed in enzymatic treated seeds. The proportion of bound TPCs to total TPCs was highest (80%) for digested raw seeds, and lowest (51%) in digested “enzymatic treated + fermented” seeds. This decrease in bound TPCs in digested samples indicates their partial conversion to extractable forms during digestion, although the quantitative changes are generally not reflected in an increase in extractable fractions.

The digestive enzymes hydrolyze starch and proteins, which may favour the release of bound phenolics. On the other hand, decreases in the content of extractable TPCs in samples after digestion are in agreement with the studies of Ortega et al. [19] and Ydjedd et al. [20], who reported a significant decrease in free phenolics after GI digestion of carob flour. Furthermore, Chait et al. [21] reported that, after the intestinal phase of digestion, the TPC values in soluble free, soluble conjugated and bound fractions of carob decreased drastically by 28%, 66%, and 68%, respectively, compared to the undigested carob sample. 

It can be concluded from these results that it is important to increase the content of extractable phenolics in seeds by bioprocessing techniques precisely because of losses during digestion. As can be seen from our research, the content of bioaccessible phenolics in bioprocessed seeds is significantly higher (up to 589% in “germinated + fermented”) than in raw seeds.

### 3.2. Phenolic Profiles

The individual extractable and bound phenolic acids obtained from raw and bioprocessed spelt seeds before and after in vitro GI digestion are shown in Table 3. Bound *trans*-ferulic acid was the most abundant phenolic compound in all digested samples, representing as much as 91% in raw seeds and only 66% in “enzymatic treated + fermented” seeds of the total concentration of all detected phenolic acids. As mentioned earlier, *trans*-ferulic acid is linked to arabinoxylans and lignin in plant cell walls in the form of covalent ester and ether linkages, and these cross-links protect cell-wall carbohydrates from microbial attack and enzymatic hydrolysis [5]. The combined effect of yeast fermentation and applied hydrolytic enzymes on spelt bran improves the extractable ferulic acid at a rate higher than spelt seeds subjected only to the fermentation process. The mixture of enzymes used in our study for the treatment of spelt seeds enables the hydrolysis of various spelt polymers, thus improving the solubility and break down of the complex cell wall structures in the bran. One of the enzymes used in our study was a feruloyl esterase, which is able to cleave the ester-bound ferulic acid of the cell-wall polymers in spelt, resulting in the release of extractable ferulic acid (unpublished results). This is in agreement with Wang et al. [5], who reported that rumen microbes are capable of breaking down the ester linkages within plant cell walls by secreting feruloyl esterase, the latter having synergistic effects with xylanase and cellulase. The breakdown of these bonds makes the cell wall more susceptible to enzymatic attack during rumen fermentation and increases cell wall degradability. On the other hand, the authors also reported that the ether bonds between ferulic acid and lignin cannot be cleaved in the rumen. Based on our results, we suggest that pretreatment of spelt seeds with external hydrolytic enzymes increases the accessibility of bound *trans*-ferulic acid for attack by degradative enzymes present in secretions of fermenting microbes. 

Among digested spelt seeds, the “germinated + fermented” seeds showed the highest content of all extractable and bound phenolic acids. A combination of germination and fermentation increased the extractable and bound *p*-coumaric, *trans*-ferulic, cis-ferulic, caffeic, and *p*-hydroxybenzoic acids by 25-fold and 10-fold, 140-fold and two -fold, 64-fold and two-fold, 30-fold and two -fold, 23-fold and two -fold, respectively, compared to digested raw seeds. It seems as if during germination an optimal quantity of spelt native enzyme was produced (or activated) when compared to external enzymatic treatment of seeds before fermentation.

After GI digestion, the contents of bound phenolic acids were decreased compared to the undigested samples. Most of the extractable phenolic acids in digested samples were also decreased, except for raw, germinated and fermented seeds. In contrast, extractable *trans*-ferulic acid was increased after digestion in “germinated + fermented” seeds by 25%, and extractable caffeic and *p*-hydroxybenzoic acids were increased in “germinated + enzymatic treated” seeds by 33% and 73%, respectively. After GI digestion, the extractable *trans*-ferulic and cis-ferulic acids content decreased strongly in some bioprocessed seeds, showing a loss in *trans*-ferulic acid, from 69% (“germinated + enzymatic treated” seeds) to 91% (enzymatic treated seeds) compared to corresponding undigested samples. Although the percentage of phenolic acids decreased after digestion is high, the content of phenolic acids in bioprocessed seeds was significantly higher than in raw seeds. It is important that our spelt seeds were bioprocessed, since large losses occur during GI digestion. Furthermore, if our bioprocessed samples had a much higher initial phenolic acids content than the raw sample, consequently, more phenolics would survive the digestion process. Among digested samples, the combinations of bioprocessed techniques had higher levels of extractable phenolic acids than raw seeds or those treated with an individual bioprocessed technique.

It is interesting that, although the extractable *trans*-ferulic acid content in the undigested seeds was 2.2–46.9-fold higher than that of extractable *p*-coumaric acid (Table 3), the content of extractable *trans*-ferulic acid in digested seeds was only 1.4–10.7-fold higher than that of extractable *p*-coumaric acid, suggesting a much higher bioaccessibility of *p*-coumaric acid than *trans*-ferulic acid. Furthermore, the same trend was also observed when we compared the contents of extractable *trans*-ferulic acid with caffeic and *p*-hydroxybenzoic acids, suggesting lower bioaccessibility of *trans*-ferulic acid than the other three phenolic acids. 

In the gastric phase, mainly the proteins present in seeds are digested and some of the phenolics bound with proteins may be released at this point. It is noteworthy that phenolics released in the gastric phase can also be labile due to the low pH (pH 3). However, some authors have previously shown that phenolic acids, such as coumaric, ferulic and caffeic acids, are absorbed from the stomach [22,23]. In the simulated digestion model performed by [24], it was shown that bound phenolics were released from wheat insoluble dietary fibre to a greater extent during the intestinal digestion stage than during the gastric digestion stage. In addition, a decrease of some phenolics in barley, chia seeds, pomegranate peel and carob flour has been reported after GI digestion [19,25]. It should be noted that, despite the decrease in the concentration of bound phenolic acids during GI digestion, their degradation remains partial. The drastic losses of phenolic acids after GI digestion are probably due to various factors: interactions with other dietary compounds, such as fibre, protein, carbohydrate; chemical reactions, mainly oxidation and polymerization, leading to the formation of other phenolic derivatives; or changes in molecular structure due to enzymatic action and, consequently, in its solubility [19]. 

In our undigested and digested raw and bioprocessed spelt seeds, compared to the bound fraction, the extractable fraction contained a number of phenolics, including gallocatechin, apigenin hexoside pentoside I, II, and III, and an unknown C-gylcosyl derivative, which were present in quite high contents (Table 4). Bioprocessing techniques significantly increased gallocatechin and C-gylcosyl derivative content in digested seeds compared to the raw seeds. However, apigenin derivatives showed a different trend in some bioprocessed seeds. 

Among digested samples, the “germinated + fermented” seeds had the highest content of all flavonoids detected, except for gallocatechin, which was the highest in “germinated + enzymatic treated” seeds. After GI digestion, compared with undigested samples, the contents of most flavonoids decreased. However, especially the unknown C-glycosyl derivative showed an increase after GI digestion in all samples, except in germinated, enzymatic treated and “fermented + enzymatic treated” seeds. Interestingly, after digestion, the raw seeds showed increased contents of apigenin I, II and C-glycosyl derivative, by 175%, 19% and 304%, respectively, compared to corresponding undigested samples. Apigenin II content also increased in digested “germinated + fermented” seeds by 2% compared to undigested sample. Gallocatechin increased by 58% after digestion only in germinated seeds. This increase in flavonoids contents may be related to hydrolysis of complex compounds from their glycoside to aglycone forms [19]. According to the research of [24], comparable amounts of wheat flavones were released in an acidic gastric environment and alkaline intestinal environment. They speculated that the increase in the amounts of flavonoids after digestion was due to the cleavage of the C-ring and reduction of double bonds.

### 3.3. Antioxidant Activity

The antioxidant activity of cereal extracts is mainly associated with their phenolics. However, the antioxidant properties of phenolics may change due to chemical transformations produced by various mechanisms during GI digestion. To evaluate the influence of GI digestion on the antioxidant activity of raw and bioprocessed spelt seeds, two assays were performed (DPPH^•^ and ABTS^•+^).

Compared to the raw seeds, bioprocessing techniques significantly increased the DPPH^•^ and ABTS^•+^ scavenging activities of extractable fractions in digested samples (Table 2). After GI digestion, the extractable DPPH values were 21-fold higher, while bound DPPH values were 2.3-fold higher in “germinated + fermented” seeds than in digested raw seeds. The same combination of bioprocessing techniques was also optimal according to the ABTS assay, but it should be stressed that the relative increase was not comparable with that of the DPPH method. Extractable and bound fraction of “germinated + fermented” seeds expressed 4.6-fold and 1.4-fold higher reactivity against ABTS^•+^, respectively. The obtained results indicate some serious quality changes in the composition of extracts under simulated in vitro digestion, since the antioxidants of the same extract showed a different response against different free radicals compared to the raw seeds. Similar variations were also observed when digested samples were compared with their undigested counterparts. For example, DPPH values of extractable phenolics of germinated seeds showed a great increase (63%) after GI digestion with reference to the undigested sample, while for ABTS, the increase was minimal (2%) for the same treatment. According to the lower reactivity of ferulic acid in the DPPH assay in comparison to the ABTS assay [4], and results presented in Table 2 and Table 3, we assume that changes in the composition of antioxidants could be related to minor unknown compounds with important antioxidant activity, which were released or formed during GI digestion. The higher antioxidant activity observed after digestion could be attributed to pH changes and deprotonation of the hydroxyl groups present on the aromatic rings of the phenolics [21]. This could also be related to the structural changes of the phenolic molecules or liberation of new compounds having higher antioxidant activity [21]. Other bioprocessed samples showed a decrease or no significant difference in extractable DPPH^•^ scavenging activity, with the highest decrease of 70% in “fermented + enzymatic treated” seeds compared to corresponding undigested seeds. This lower activity could be due to the lower TPC and/or the fact that some phenolics can be converted into different structural forms with different chemical properties, due to their sensitivity to neutral pH [20]. According to Rice-Evans et al. [26], the chemical structure of phenolics also plays a role in free radical-scavenging activity, which mainly depends on the number and position of hydrogen-donating hydroxyl groups on the aromatic rings of phenolic molecules. The DPPH assay may involve electron transfer reactions and acids and alkalis present in extracts may affect the ionization equilibrium of phenolics, leading to a reduction or enhancement of the reaction rate [27]. In addition to possible intermolecular interactions (hydrogen bonds) of phenolic acids with the solvent in the reaction mixture, which can decrease their reactivity in the antioxidant assay, phenolic acids can also form intramolecular hydrogen interactions, which may also affect the transfer of a hydrogen atom to a free radical [28]. 

All antioxidant activities of bound fractions tested decreased strongly after digestion in comparison to the undigested seeds. This reduction in activity could be due to the decrease in bound TPCs after digestion. The antioxidant activity is tightly related to phenolics content and composition. Similarly, Correa-Betanzo et al. [29] showed that DPPH^•^ scavenging activity of blueberry extracts decreased over 50% after intestinal digestion. Chen et al. [30] also reported that the DPPH values of different sesame varieties were significantly lower after digestion than before digestion. On the other hand, Chait et al. [21] reported that DPPH and ABTS levels of carob phenolics in soluble free form showed a significant increase to 107 mg Gallic equivalents (GAE)/g and 399 mg TE/g, respectively, under GI digestion compared to undigested extract. 

Coefficients of correlation (r) were calculated to explain the relationship between TPCs of extractable and bound fractions and their antioxidant activities (DPPH^•^ and ABTS^•+^). Before and after GI digestion, the extractable fraction showed a positive and strong correlation between TPC and the antioxidant activity measured with DPPH (*r* = 0.923 and *r* = 0.978, respectively) and ABTS (*r* = 0.918 and *r* = 0.973, respectively) assays. Furthermore, before and after GI digestion, the DPPH (*r* = 0.951 and *r* = 0.911, respectively) and ABTS (*r* = 0.959 and *r* = 0.945, respectively) values found in bound fractions were also strongly positively correlated with TPC. These results agree with several previous studies [21,31], which reported a high correlation between TPC and antioxidant activities. Thus, these results indicate that phenolics widely contributed to the antioxidant activity of spelt seeds. Moreover, the antioxidant activity of phenolics depends essentially on their molecular structure. It has been shown that the CH=CH-COOH grouping of hydroxycinnamic acids ensures a greater capacity to transfer a proton and subsequently to stabilize radicals than the carboxyl (COOH) grouping of hydroxybenzoic acids. In addition, flavonoids can act as proton or electron donors, which also lead to a good correlation with them. Cereal extracts are very complex mixtures of many different compounds with different activities. The different activity may be due to the synergistic or antagonistic action of these compounds [26].

The reduction in antioxidant activity (DPPH^•^ and ABTS^•+^) observed in our study and in the work of other authors during the intestinal part of in vitro digestion can be explained by the structural reorganisation of some bioactive compounds when the pH is changed to slightly alkaline. Moreover, these compounds gain the ability to react and bind with other components of the seed matrix, leading to a decrease in their antioxidant activity [32]. 

### 3.4. Bioaccessibility and Biostability of Digested Phenolics

#### 3.4.1. Bioaccessibility and Biostability of Total Phenolics Content in Analysed Spelt Seeds

**Figure 1:**
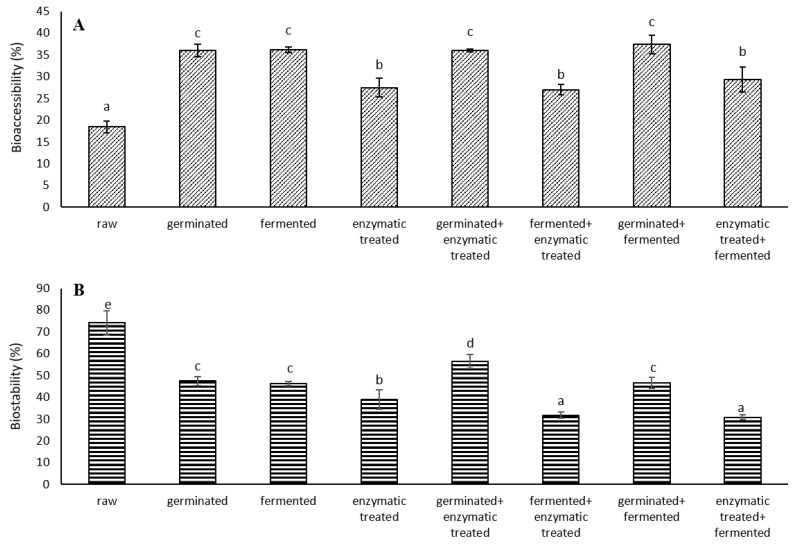
(**A**) Bioaccessibility and (**B**) biostability of total phenolic content (TPC) from raw and bioprocessed spelt seeds after in vitro digestion. The percentages were calculated with respect to corresponding undigested spelt seed TPCs. Results were expressed as the mean of three replicates and the error bars indicate standard deviation. Different letters indicate significant differences (*p* < 0.05; Duncan’s Multiple Range Test).

After in vitro digestion, part of the phenolics from spelt seeds were released into the digestion supernatant, indicating that they became bioaccessible and could be absorbed by our body. The bioaccessibility of TPCs of the analysed spelt seeds was evaluated after GI digestion by Folin-Ciocalteu assay compared to corresponding undigested samples. Figure 1A shows the percentage of TPCs bioaccessibility for all samples, estimated by GI digestion. The seeds that showed the highest bioaccessibility were “germinated + fermented” (37.4%), followed by fermented, germinated and “germinated + enzymatic treated”. These samples also showed higher extractable TPCs than other digested samples; furthermore, “germinated + fermented” seeds also showed the highest content of extractable phenolic acids and contained most flavonoids after digestion. The raw seeds showed the lowest bioaccessibility (18.4%). It was shown that bioprocessing of spelt seeds increases the bioaccessibility of phenolics by 47% (“fermented + enzymatic treated”) to 103% (“germinated + fermented”) compared to raw seeds. The great increase in bioaccessibility in bioprocessed seeds may be due to the hydrolysis of different spelt fibre polymers by hydrolytic enzymes, which may lead to a structural degradation of bran cell walls [33]. The lower bioaccessibility of phenolics in raw seeds may be due to the fact that most phenolics in plants are conjugated to other molecules, such as carbohydrates, cellulose, and lignin, which are resistant to digestion.

Part of the phenolics remained in the digested spelt seeds residue, indicating that they are not bioaccessible (bound phenolics). We further examined the biostability of these phenolics (Figure 1B). In raw seeds, a significantly higher biostability of phenolics was observed compared to bioprocessed seeds. The biostability of TPC in raw seeds was from 31% to 141% higher than in other bioprocessed seeds. The lowest biostability of TPC was seen for “enzymatic treated + fermented” and “fermented + enzymatic treated” seeds. Among bioprocessed seeds, the highest biostability of TPC was observed for “germinated + enzymatic treated” seeds. Since the percentage of biostability is calculated based on the total (extractable + bound) phenolics of the undigested samples, the biostability of raw seeds was consequently higher, since the content of extractable phenolics is significantly higher in bioprocessed seeds. Although raw seeds had the highest percentage of phenolics biostability, the content of biostable phenolics in the “germinated + fermented” seeds was significantly higher (2-fold) than in raw seeds. The more stable the phenolics are during GI digestion, the more they continue to enter the colon, where the microbiota metabolizes phenolics into products that can be absorbed by our body. However, Ren et al. [34] reported that most phenolics with multiple hydroxyl groups are highly unstable. One of the ways of improving the bioaccessibility or biostability of phenolics is encapsulation of active ingredients.

#### 3.4.2. Bioaccesebility and Biostability of Individual Phenolics in Analysed Spelt Seeds

The bioaccessibility of individual phenolic acids and flavonoids is shown in Table 5. Bioprocessing of spelt seeds increased the bioaccessibility of phenolic acids compared to raw seeds. The bioaccessibility of phenolic acids varied greatly among differently treated spelt seeds. 

Bioprocessing of spelt seeds by fermentation and enzymatic treatment, alone or in combination, showed the highest bioaccessibility of *p*-coumaric acid. However, the amount of released (extractable) *p*-coumaric acid after digestion was the highest in “germinated + fermented” seeds, although the proportion of extractable form against the bound form was completely different in these spelt seeds. The most effective bioprocessing techniques were their combinations, which increased *trans*-ferulic acid bioaccessibility by 24–63-fold compared to raw seeds. Similar results were also observed by Anson et al. [2], who reported that the most effective bioprocessing technique was a combination of fermentation and enzymatic treatment of wheat bran, which increased bioaccessibility of ferulic acid by 5-fold compared to native bran. Bioprocessing techniques resulted in an increase in cis-ferulic acid bioaccessibility from 2.8-fold in fermented seeds to 22-fold in “germinated + fermented” seeds compared to raw seeds. The bioaccessibility of caffeic acid increased from 2.7-fold in enzymatic treated seeds to 6.6-fold in “germinated + fermented” seeds and the bioaccessibility of *p*-hydroxybenzoic acid increased from 1.2-fold in enzymatic treated seeds to 3.4-fold in germinated seeds compared to raw seeds. Among phenolic acids, *p*-hydroxybenzoic acid showed the highest bioaccessibility, from 16.5% (raw seeds) to 56.8% (germinated seeds). The proportion of extractable *p*-hydroxybenzoic acid over total (extractable + bound) *p*-hydroxybenzoic acid was drastically higher than in case of other phenolic acids, ranging from 17% to 87%. It is interesting that the digested “germinated + fermented” seeds, which showed the highest content of bioaccessible *p*-coumaric, *trans*-ferulic and *p*-hydroxybenzoic acids, did not show the highest percentage of bioaccessibility. The reason was the content of extractable and bound phenolic acids in undigested seeds. In undigested “germinated + fermented” seeds, the content of bound *p*-coumaric and *trans*-ferulic acids, as well as the content of extractable *p*-hydroxybenzoic acid, was significantly higher than in other undigested samples, which we considered when calculating bioaccessibility.

Phenolics that stay in the digested residue (bound phenolics) and are not released into the digestive system are biostable. Several authors pointed out that the stability of phenolics during the GI digestion process is strongly influenced by their chemical structure, as phenolics have different sensitivities to pH variations and digestive enzymes activity [35,36]. The biostability or, as some authors call it, the recovery of phenolic acids, is presented in Table 5. In the digestion residue, the highest percentage of phenolic acids remained biostable in raw seeds, which is consistent with TPC biostability. This is quite logical, since the raw seeds are not pre-treated with bioprocessing techniques that make the material more accessible to digestive enzymes. Consequently, raw seeds have the highest proportion of phenolics bound to various components of the cell wall. Sęczyk et al. [37] suggested that food matrices with a high content of insoluble dietary fibre and proteins have stronger interactions with phenolics and lower digestibility. Xu et al. [38] reported that non-starch polysaccharides protect the phenolics bound to them from enzymes in the mouth, stomach and small intestine. When the non-starch polysaccharides enter the colon, they can be digested by the colon microbiota, releasing phenolics that protect us against colon cancer. In general, *p*-coumaric and *trans*-ferulic acids showed the highest biostability, while *p*-hydroxybenzoic acid showed the lowest biostability. Tomé-Sánchez et al. [17] also reported that ferulic acid derivatives of barley and wheat sprouts have high stability to digestion conditions. Interestingly, the lowest biostability of *p*-hydroxybenzoic acid was found in “enzymatic treated + fermented” seeds; it was almost 10-fold lower than in raw seeds. However, the bioaccessibility of *p*-hydroxybenzoic acid was significantly higher in “enzymatic treated + fermented” seeds than in raw seeds, so it could be suggested that some of the bound *p*-hydroxybenzoic acid was released from food matrix into the digestive fluid during digestion and may also change its structure to form new derivatives. A similar trend was also observed for other phenolic acids. 

Despite the substantial increase in the bioaccessibility of phenolic acids achieved by bioprocessing, the major part of the phenolic acids remained in the non-bioaccessible (biostable) fraction that will enter the colon. Fermentation of cell-wall structures in the colon by bacterial enzymes is expected to facilitate the release of phenolic acids that were not accessible in the small intestine. It is therefore important to understand how the digestion process affects the phenolic structure and stability, since this, in turn, influences bioaccessibility of phenolics and their potential beneficial effects in cells of the gut epithelium [29]. 

As reported by Koistinen et al. [7], the bioaccessibility of ferulic acid was significantly higher in bioprocessed rye bread (yeast fermentation + enzymatic treatment) than in non-bioprocessed rye bread (88% vs. 51%, respectively). Gullon et al. [39] also reported that flavonoid bioaccessibility (64%) was higher than that of phenolics (36%). Kroon et al. [40] also indicated that only 2.6% of ferulic acid was released from wheat under GI digestion. Hemery et al. [41] reported that in whole-grain and bran-rich breads, the bioaccessibility ranged from 6% to 15% for *p*-coumaric acid, and from 2.5% to 5% for ferulic acid. The above data are difficult to compare directly, because to the best of our knowledge, studies about the effects of digestion on bioaccessibility often use different calculations for bioaccessibility or biostability. 

The increase in extractable flavonoids in undigested spelt seeds caused by bioprocessing was not necessarily reflected in an increase in bioaccessible flavonoids during digestion of samples (Table 5). The highest content of bioaccessible gallocatechin had “germinated + enzymatic treated” seeds and during digestion there was no decrease. Furthermore, germinated seeds showed the highest increase in gallocatechin after digestion and, consequently, the bioaccessibility was also the highest. Enzymatic treated seeds showed the lowest bioaccessibility of gallocatechin, which is probably due to the fact that gallocatechin content decreased significantly after digestion (by 37%). Interestingly, apigenin II and III showed the highest bioaccessibility in raw seeds, probably due to the greater proportion of the extractable form, which increased significantly after digestion (by 175% and 19%, respectively). The bioprocessed seeds showed significantly lower bioaccessibility of apigenin II and III, although germination, alone or in combination, showed significantly higher bioaccessible apigenin II content and “germination + fermentation” showed significantly higher apigenin III content. The highest bioaccessibility of apigenin I and an unknown C-glycosyl derivative was found in “germinated + fermented” seeds. The lowest bioaccessibility of apigenin I was found in enzymatic treated seeds, which also showed the highest decrease in apigenin I content after digestion (50%). The lowest bioaccessibility of C-glycosyl derivative was found in germinated seeds. 

The highest biostability of gallocatechin, apigenin II and C-glycosyl derivative was found in raw seeds. The highest biostability of apigenin I and III was found in “germinated + enzymatic treated” seeds and germinated seeds, respectively. It is interesting to note that, among tested flavonoids, the C-glycosyl derivative showed the highest biostability; it was more than 65%. Furthermore, the C-glycosyl derivative was only up to 26% bioaccessible. On the other hand, gallocatechin generally showed the lowest biostability.

The above results suggest that during GI digestion of spelt seeds, various changes may have occurred in phenolic acids and flavonoids, such as (i) a change in chemical structure, (ii) increased or decreased solubility, or (iii) interaction with other compounds, affecting their bioaccessibility [39]. Adom and Liu [42] reported that insoluble bound phenolics could resist GI digestion to reach the colon, because cell wall components are difficult to digest. The higher fibre and protein content could partially prevent digestive enzymes from releasing the bound phenolics, thus limiting their bioaccessibility. Furthermore, β-glucan is a soluble, viscous dietary fibre that may contribute to the low phenolics bioaccessibility, since ß-glucans can form viscous gels that can trap phytochemicals, including phenolics. This may also explain part of the particularly lower bioaccessibility in the spelt seeds found in this study, since Triticum bran is a good source of dietary fibre, consisting mainly of arabinoxylans, cellulose, and β-glucan [33].

Zeng et al. [43] reported that although wheat possessed the highest TPC and antioxidant activity, the bioaccessible phenolics were lower than those of brown rice and oat. This suggests that cereals with the most abundant phenolics are not necessarily those with the highest bioaccessibility. The cereal matrix appears to be a crucial factor in their digestibility and stability during digestion, affecting bioaccessibility. Although ferulic acid is the most abundant phenolic compound in spelt, the bioaccessibility of ferulic acid (up to 6.3%) was significantly lower than that of TPC (up to 37.4%). A possible reason for this large discrepancy was that many of the compounds present in the supernatant of the digested samples were unknown. 

### 3.5. Pearson Correlation Analysis

#### 3.5.1. Correlations between the Proportion of Extractable Phenolics and Bioaccessibility

There were large differences in the bioaccessibility of phenolic acids in different spelt seeds. The correlation between the proportion of extractable *p*-coumaric (*r* = 0.997), *trans*-ferulic (*r* = 0.942), cis-ferulic (*r* = 0.960) and caffeic (*r* = 0.976) acids over the total (extractable + bound) phenolic acid in the digested samples and the bioaccessibility (refers to undigested samples) was very strong, while the correlation between the proportion of extractable *p*-hydroxybenzoic acid and its bioaccessibility was less strong (*r* = 0.737). Interestingly, the proportion of extractable TPC over the total TPC in digested samples correlated only moderately (*r* = 0.653) with TPC bioaccessibility. The lower correlation between extractable TPC and bioaccessibility was probably also influenced by some flavonoids that showed moderate correlation with bioaccessibility. The proportion of gallocatechin (*r* = 0.687), apigenin I (*r* = 0.538), III (*r* = 0.536) over the total of digested samples correlated moderately with their bioaccessibility, while apigenin II (*r* = 0.883) and C-glycosyl derivative (*r* = 0.956) correlated strongly with their bioaccessibility. It can therefore be concluded that the increase in bioaccessibility of hydroxycinnamic acids might be related to the increase in the proportion of extractable phenolic acids in spelt seeds. Mateo Anson et al. [44] also demonstrated a strong correlation between the proportion of free ferulic acid and bioaccessibility among five breads.

#### 3.5.2. Correlations between Extractable Phenolic Acids of Digested and Undigested Spelt Seeds

The correlations between the amounts of extractable phenolic acids in digested spelt seeds and the amounts of extractable phenolic acids in the corresponding undigested seeds were calculated. The r-values were 0.966, 0.946, 0.938, 0.706 and 0.445 for extractable caffeic, *p*-coumaric, *p*-hydroxybenzoic, cis-ferulic and *trans*-ferulic acids, respectively. The strong positive correlation suggests that the higher level of caffeic, *p*-coumaric and *p*-hydroxybenzoic acids in undigested spelt seeds results in a higher level of bioaccessible caffeic, *p*-coumaric and *p*-hydroxybenzoic acids. Meanwhile, the correlation between extractable *trans*-ferulic acid in undigested and digested samples was moderately positive. This suggests that, although spelt seeds could be considered as a ferulic acid-rich source, a higher extractable ferulic acid content before digestion is not necessarily associated with a higher content after digestion.

### 3.6. Principal Component Analysis

**Figure 2:**
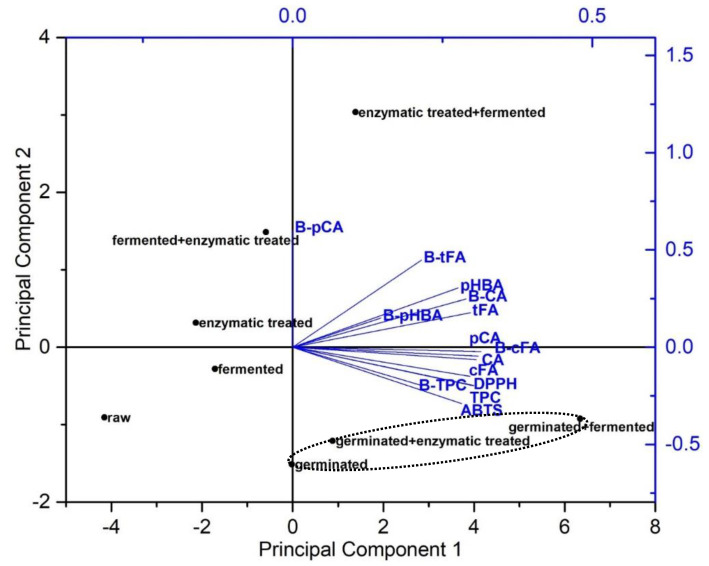
Principal component analysis (PCA) biplot of content of bioaccessible *p*-coumaric (pCA), *trans*-ferulic (tFA), *cis*-ferulic (cFA), caffeic (CA) and *p*-hydroxybenzoic (pHBA) acids, total phenolic content (TPC), scavenging activities against DPPH^•^ (DPPH) and ABTS^•+^ (ABTS) radicals and bioaccessibility of *p*-coumaric acid (B-pCA), *trans*-ferulic acid (B-tFA), *cis*-ferulic acid (B-cFA), caffeic acid (B-CA), *p*-hydroxybenzoic acid (B-pHBA) and total phenolic content (B-TPC) of raw and bioprocessed spelt seeds.

PCA (Figure 2) was used to explore similarities among the quantities of total and individual phenolics and their antioxidant activities in the bioaccessible fraction of spelt seeds and their percentage of bioaccessibility. Principal component 1 (PC1) corresponded to 69.4% of the data variation and differentiated spelt seeds according to the amounts of *p*-coumaric, *trans*-ferulic, *cis*-ferulic, and caffeic acids, bioaccessibility of *cis*-ferulic and caffeic acids, TPC and its bioaccessibility, and DPPH assay. PC2 explained 17.4% of the data variance due the amounts of *p*-hydroxybenzoic acid and its bioaccessibility, bioaccessibility of *p*-coumaric and *trans*-ferulic acids, and ABTS assay. Notably, germination, alone or in combination, showed the highest bioaccessible TPC and its percentage of bioaccessibility (bioaccessible TPC/totals in undigested sample), as well as the highest antioxidant activity against DPPH^•^ and ABTS^•+^ and content of bioaccessible cis-ferulic and caffeic acids. More specifically, “germinated + fermented” seeds showed the highest content of bioaccessible TPC, individual phenolic acids and their antioxidant activities. Interestingly, “enzymatic treated + fermented” seeds are positioned in the right upper side of the graph and showed the highest percentage of bioaccessibility of *p*-coumaric and *trans*-ferulic acids, while the content of these bioaccessible phenolic acids was significantly higher in “germinated + fermented” seeds (108% and 55%, respectively). “Fermented + enzymatic treated” seeds were distinguished by a high bioaccessibility of *p*-coumaric and *trans*-ferulic acids. Enzymatic treated seeds were characterised by higher bioaccessibility of *p*-coumaric acid. Raw seeds are positioned in the lower left side of the graph and showed the lowest content of bioaccessible TPC, individual phenolic acids and their antioxidant activities, as well as showing the lowest percentage of bioaccessibility of TPC and individual phenolic acids.

Overall, the results presented here showed that the bioprocessing of spelt seeds significantly improved the bioaccessibility of TPC and individual phenolic acids compared to raw seeds. In general, the combination of germination and fermentation was the most effective bioprocessing technique, showing the highest content of total and individual bioaccessible phenolics and higher bioaccessibility than other digested samples. 

## 4. Conclusions

Bioprocessing techniques aimed at improving the bioaccessibility of phenolics from cereal products may represent the most promising approach to improving health benefits at the systemic level. The study showed a statistically significant effect of bioprocessing techniques on the content of bioaccessible TPC, individual phenolics and their antioxidant activities. Raw seeds showed the lowest content of bioaccessible TPC and individual phenolics, as well as their antioxidant activities. A combination of bioprocessing techniques, especially “germination + fermentation”, was the most effective method to drastically improve the content of total and individual bioaccessible phenolics and their antioxidant activities. However, bioaccessibility of *p*-coumaric and *trans*-ferulic acids was the highest in “enzymatic treated + fermented” seeds. The application of the in vitro digestion model allowed a more detailed characterisation of the content of individual phenolics in spelt seeds that are actually accessible to the human body. However, in cereals, most phenolics are in bound form and may not be released during GI digestion but only in the colon. We should therefore highlight a problem that exists when using only GI digestion, namely the lack of a fermentation stage in the large intestine, where phenolics are highly metabolized. We do not therefore have a complete picture of the bioaccessibility of phenolics in the human body.

## Figures and Tables

**Table 1:** Composition of stock solutions of simulated digestion fluids. SSF: simulated salivary fluid, SGF: simulated gastric fluid, and SIF: simulated intestinal fluid.

		SSF (pH 7)		SGF (pH 3)		SIF (pH 7)	
Salt Solution	Stock Concentration (mol/L)	mL of Stock Added to Prepare 0.4 L (mL)	Final Salt Concentration in SSF (mmol/L)	mL of Stock Added to Prepare 0.5 L (mL)	Final Salt Concentration in SGF (mmol/L)	mL of Stock Added to Prepare 1 L (mL)	Final Salt Concentration in SIF (mmol/L)
KCl	0.5	15.1	15.1	8.6	6.9	17	6.8
KH_2_PO_4_	0.5	3.7	3.7	1.1	0.9	2	0.8
NaHCO_3_	1	6.8	13.6	15.6	25	106.3	85
NaCl	2	/	/	14.8	47.2	24	38.4
MgCl_2_(H_2_O)_6_	0.15	0.5	0.15	0.5	0.12	2.8	0.33
(NH_4_)_2_CO_3_	0.5	0.06	0.06	0.6	0.5	/	/
CaCl_2_(H_2_O)_2_	0.3	/	1.50	/	0.15	/	0.6

**Table 2:** Total phenolic content (TPC) and scavenging activities against DPPH^•^ [DPPH] and ABTS^•+^ [ABTS] radicals of undigested and digested spelt seeds in extractable and bound fractions.

	TPC		DPPH		ABTS	
	mg TE/g DW		mg TE/g DW		mg TE/g DW	
Sample	Extractable	Bound	Extractable	Bound	Extractable	Bound
***Undigested***						
raw	1.28 ^a^	6.73 ^D^	0.04 ^a^	0.85 ^EF^	1.30 ^a^	6.98 ^F^
germinated	4.85 ^e^	10.76 ^G^	0.43 ^d^	1.50 ^H^	4.21 ^d^	8.90 ^HI^
fermented	3.89 ^c^	7.77 ^E^	0.26 ^b^	0.87 ^F^	4.96 ^e^	7.13 ^F^
enzymatic treated	4.52 ^de^	6.13 ^C^	0.89 ^g^	0.80 ^E^	6.31 ^f^	6.01 ^E^
germinated + enzymatic treated	7.29 ^h^	9.90 ^F^	0.91 ^g^	1.69 ^J^	6.97 ^g^	8.14 ^G^
fermented + enzymatic treated	8.57 ^i^	5.72 ^BC^	1.21 ^i^	0.79 ^E^	7.45 ^h^	5.61 ^CD^
germinated + fermented	14.19 ^k^	13.08 ^H^	1.57 ^k^	1.84 ^K^	9.61 ^i^	9.05 ^I^
enzymatic treated +fermented	8.02 ^i^	6.10 ^C^	1.15 ^h^	0.85 ^EF^	7.16 ^g^	5.83 ^DE^
***Digested***						
raw	1.48 ^a^	5.98 ^BC^	0.06 ^a^	0.70 ^D^	1.35 ^a^	6.10 ^E^
germinated	5.62 ^f^	7.39 ^E^	0.70 ^e^	0.40 ^B^	4.28 ^d^	7.21 ^F^
fermented	4.22 ^cd^	5.39 ^B^	0.23 ^b^	0.35 ^AB^	2.33 ^c^	6.09 ^E^
enzymatic treated	2.92 ^b^	4.13 ^A^	0.24 ^b^	0.56 ^C^	1.94 ^b^	5.36 ^BC^
germinated + enzymatic treated	6.19 ^g^	9.71 ^F^	0.75 ^f^	1.32 ^G^	4.28 ^d^	7.26 ^F^
fermented + enzymatic treated	3.85 ^c^	4.53 ^A^	0.36 ^c^	0.36 ^AB^	2.28 ^c^	5.15 ^B^
germinated + fermented	10.19 ^j^	12.48 ^H^	1.28 ^j^	1.60 ^I^	6.24 ^f^	8.57 ^H^
enzymatic treated + fermented	4.14 ^c^	4.34 ^A^	0.44 ^d^	0.31 ^A^	2.37 ^c^	4.51 ^A^

Results were expressed as the mean of three replicates. Means with different small letters within a column indicate significant difference between the extractable phenolic contents (*p* < 0.05; Duncan’s multiple range test). Means with different capital letters within a column indicate significant difference between the bound phenolic contents (*p* < 0.05; Duncan’s multiple range test).

**Table 3:** Contents of individual phenolic acids of undigested and digested spelt seeds in extractable and bound fractions.

	Phenolic Acids (µg/g DW)									
Sample	*p*-Coumaric Acid		*trans*-Ferulic Acid		*cis*-Ferulic Acid		Caffeic Acid		*p*-Hydroxybenzoic Acid	
	Extractable	Bound	Extractable	Bound	Extractable	Bound	Extractable	Bound	Extractable	Bound
***Undigested***										
raw	0.27 ^a^	28.86 ^A^	0.59 ^a^	834.99 ^E^	0.30 ^a^	37.14 ^E^	0.29 ^a^	7.87 ^E^	1.20 ^a^	12.57 ^B^
germinated	3.54 ^d^	221.60 ^E^	9.19 ^bc^	1466.45 ^J^	4.75 ^c^	80.77 ^K^	2.92 ^e^	13.98 ^I^	14.24 ^f^	18.13 ^D^
fermented	1.09 ^b^	32.11 ^BC^	3.58 ^a^	965.96 ^G^	1.56 ^b^	54.02 ^G^	0.90 ^b^	5.17 ^BC^	9.21 ^c^	15.30 ^C^
enzymatic treated	6.37 ^h^	36.07 ^C^	140.68 ^j^	578.91 ^E^	8.14 ^f^	37.86 ^E^	5.06 ^h^	8.46 ^E^	24.81 ^i^	30.12 ^EF^
germinated + enzymatic treated	7.90 ^j^	265.04 ^G^	111.56 ^i^	1351.42 ^I^	7.62 ^ef^	61.57 ^H^	3.93 ^g^	14.48 ^I^	12.19 ^de^	49.63 ^G^
fermented + enzymatic treated	7.95 ^j^	38.21 ^C^	345.74 ^l^	532.01 ^CD^	22.87 ^h^	26.87 ^B^	7.63 ^j^	6.39 ^D^	36.29 ^j^	29.62 ^E^
germinated + fermented	15.31 ^l^	298.17 ^H^	68.32 ^g^	1699.14 ^K^	30.84 ^i^	77.15 ^J^	22.56 ^l^	14.40 ^I^	97.27 ^m^	31.50 ^EF^
enzymatic treated + fermented	6.84 ^i^	35.67 ^C^	320.92 ^k^	549.15 ^DE^	19.17 ^g^	30.38 ^C^	6.42 ^i^	6.30 ^D^	70.08 ^l^	29.34 ^E^
***Digested***										
raw	0.44 ^a^	28.07 ^A^	0.61 ^a^	820.05 ^F^	0.49 ^a^	33.27 ^D^	0.53 ^a^	7.59 ^E^	2.27 ^b^	11.18 ^B^
germinated	4.06 ^e^	185.19 ^D^	14.39 ^c^	1210.44 ^H^	6.36 ^d^	44.52 ^F^	3.27 ^f^	9.19 ^F^	18.39 ^g^	17.60 ^D^
fermented	2.25 ^c^	27.80 ^A^	4.26 ^ab^	805.45 ^F^	2.05 ^b^	33.76 ^D^	1.35 ^c^	2.61 ^A^	11.57 ^d^	10.48 ^B^
enzymatic treated	3.69 ^d^	33.56 ^BC^	12.48 ^c^	493.10 ^B^	2.10 ^b^	28.01 ^BC^	2.40 ^d^	8.65 ^EF^	11.26 ^d^	16.62 ^CD^
germinated + enzymatic treated	5.70 ^g^	238.38 ^F^	34.95 ^d^	1209.11 ^H^	7.26 ^e^	55.35 ^G^	5.24 ^h^	11.05 ^G^	21.03 ^h^	29.64 ^E^
fermented + enzymatic treated	3.90 ^de^	39.17 ^C^	41.55 ^e^	508.46 ^BC^	2.04 ^b^	21.90 ^A^	3.73 ^g^	5.73 ^C^	26.82 ^i^	9.73 ^AB^
germinated + fermented	11.05 ^k^	293.85 ^H^	85.61 ^h^	1668.17 ^K^	31.45 ^i^	65.84 ^I^	15.93 ^k^	12.84 ^H^	52.57 ^k^	31.75 ^F^
enzymatic treated + fermented	5.32 ^f^	36.29 ^C^	55.21 ^f^	382.21 ^A^	6.31 ^d^	22.23 ^A^	5.06 ^h^	4.95 ^B^	53.46 ^k^	8.30 ^A^

Data are means (*n* = 6). Means with different small letters within a column (phenolic acid) indicate significant difference between the extractable phenolic contents (*p* < 0.05; Duncan’s multiple range test). Means with different capital letters within a column indicated significant difference between the bound phenolic contents (*p* < 0.05; Duncan’s multiple range test).

**Table 4:** Contents of individual flavonoids of undigested and digested spelt seeds in extractable fraction.

	Flavonoids (µg/g DW)				
	Gallocatechin	Apigenin			Unknown C-Glycosyl Derivative
		I	II	III	
Sample	Extractable	Extractable	Extractable	Extractable	Extractable
***Undigested***					
raw	1.01 ± 0.13 ^a^	4.28 ± 0.06 ^a^	1.36 ± 0.04 ^a^	8.95 ± 0.29 ^d^	0.45 ± 0.03 ^a^
germinated	7.16 ± 0.28 ^e^	6.17 ± 0.13 ^d^	7.72 ± 0.30 ^i^	7.21 ± 0.29 ^b^	2.86 ± 0.16 ^e^
fermented	3.42 ± 0.13 ^b^	5.88 ± 0.15 ^d^	2.36 ± 0.14 ^c^	9.70 ± 0.29 ^e^	1.32 ± 0.03 ^b^
enzymatic treated	6.24 ± 0.26 ^d^	8.47 ± 0.23 ^g^	6.02 ± 0.19 ^g^	13.02 ± 0.40 ^g^	4.82 ± 0.11 ^j^
germinated + enzymatic treated	31.21 ± 1.48 ^l^	7.56 ± 0.22 ^f^	7.33 ± 0.25 ^h^	10.69 ± 0.27 ^f^	4.70 ± 0.12 ^j^
fermented + enzymatic treated	22.95 ± 0.59 ^i^	9.85 ± 0.28 ^i^	5.03 ± 0.22 ^f^	15.28 ± 0.31 ^h^	4.04 ± 0.10 ^i^
germinated + fermented	39.57 ± 1.00 ^m^	23.21 ± 0.57 ^k^	18.95 ± 0.68 ^j^	35.19 ± 0.95 ^j^	7.13 ± 0.37 ^k^
enzymatic treated + fermented	24.37 ± 0.69 ^j^	9.23 ± 0.38 ^h^	5.17 ± 0.15 ^f^	15.67 ± 0.25 ^h^	2.11 ± 0.13 ^d^
***Digested***					
raw	1.00 ± 0.17 ^a^	4.33 ± 0.06 ^a^	3.74 ± 0.20 ^e^	10.66 ± 0.15 ^f^	1.82 ± 0.04 ^c^
germinated	11.34 ± 0.22 ^f^	4.78 ± 0.16 ^b^	5.92 ± 0.52 ^g^	5.98 ± 0.30 ^a^	2.83 ± 0.14 ^e^
fermented	3.60 ± 0.07 ^bc^	4.26 ± 0.31 ^a^	1.90 ± 0.05 ^b^	7.91 ± 0.51 ^c^	3.37 ± 0.09 ^g^
enzymatic treated	3.93 ± 0.27 ^c^	4.27 ± 0.18 ^a^	2.11 ± 0.09 ^bc^	8.03 ± 0.41 ^c^	3.44 ± 0.11 ^g^
germinated + enzymatic treated	32.13 ± 0.84 ^k^	7.49 ± 0.71 ^f^	7.58 ± 0.34 ^hi^	7.75 ± 0.50 ^c^	8.20 ± 0.21 ^l^
fermented + enzymatic treated	20.93 ± 0.92 ^h^	6.93 ± 0.61 ^e^	2.82 ± 0.18 ^d^	7.78 ± 0.44 ^c^	3.72 ± 0.22 ^h^
germinated + fermented	25.89 ± 0.43 ^k^	18.19 ± 0.75 ^j^	19.27 ± 0.57 ^k^	16.83 ± 0.82 ^i^	10.62 ± 0.38 ^m^
enzymatic treated + fermented	17.51 ± 0.81 ^g^	5.44 ± 0.54 ^c^	2.84 ± 0.16 ^d^	9.79 ± 0.35 ^e^	3.14 ± 0.16 ^f^

Data are means ± standard deviation. Means with different letters within the same column indicated significant difference between the extractable flavonoid contents (*p* < 0.05; Duncan’s multiple range test).

**Table 5:** Percentages of bioaccessibility and biostability for individual phenolics from raw and bioprocessed spelt seeds after in vitro digestion.

	Bioaccessibility (%)									
Digested Spelt Seeds	*p*-Coumaric Acid	*trans*-Ferulic Acid	*cis*-Ferulic Acid	Caffeic Acid	*p*-Hydroxybenzoic Acid	Gallocatechin	Apigenin			Unknown C-glycosyl Derivative
							I	II	III	
raw	1.5 ^a^	0.1 ^a^	1.3 ^a^	6.5 ^a^	16.5 ^a^	43.1 ^a^	45.6 ^e^	83.4 ^g^	61.0 ^e^	10.9 ^b^
germinated	1.8 ^b^	1.0 ^c^	7.4 ^e^	19.3 ^c^	56.8 ^g^	85.9 ^f^	32.5 ^b^	32.7 ^b^	33.5 ^a^	8.1 ^a^
fermented	6.8 ^e^	0.4 ^b^	3.7 ^b^	22.2 ^d^	47.2 ^e^	66.9 ^d^	39.1 ^d^	36.2 ^d^	44.2 ^d^	22.8 ^f^
enzymatic treated	8.7 ^f^	1.7 ^d^	4.6 ^d^	17.8 ^b^	20.5 ^b^	41.2 ^a^	30.4 ^a^	21.4 ^a^	37.6 ^b^	16.8 ^c^
germinated + enzymatic treated	2.1 ^c^	2.4 ^e^	10.5 ^f^	28.5 ^f^	34.0 ^c^	85.3 ^f^	47.2 ^f^	45.5 ^e^	35.5 ^b^	20.1 ^e^
fermented + enzymatic treated	8.5 ^f^	4.7 ^f^	4.1 ^c^	26.6 ^e^	40.7 ^d^	78.6 ^e^	47.8 ^f^	34.9 ^c^	34.9 ^a^	21.7 ^f^
germinated + fermented	3.5 ^d^	4.8 ^f^	29.1 ^h^	43.1 ^h^	40.8 ^d^	58.1 ^b^	61.5 ^g^	69.8 ^f^	36.6 ^b^	26.0 ^g^
enzymatic treated + fermented	12.5 ^g^	6.3 ^g^	12.7 ^g^	39.8 ^g^	53.8 ^f^	61.5 ^c^	36.4 ^c^	30.6 ^b^	41.2 ^c^	17.4 ^d^
	**Biostability (%)**									
raw	96.4 ^f^	98.1 ^f^	88.9 ^e^	93.1 ^h^	81.2 ^h^	48.4 ^f^	48.3 ^e^	53.0 ^h^	42.7 ^e^	91.7 ^e^
germinated	82.3 ^b^	82.0 ^d^	52.1 ^b^	54.4 ^e^	54.4 ^g^	37.0 ^e^	48.5 ^e^	40.9 ^e^	59.3 ^g^	89.0 ^d^
fermented	83.7 ^b^	83.1 ^d^	60.8 ^c^	43.0 ^d^	42.8 ^e^	29.4 ^c^	13.8 ^a^	43.7 ^f^	29.9 ^b^	80.9 ^c^
enzymatic treated	79.1 ^a^	68.5 ^c^	60.9 ^c^	64.0 ^f^	30.2 ^d^	35.5 ^d^	39.2 ^d^	37.1 ^d^	38.4 ^d^	74.6 ^b^
germinated + enzymatic treated	87.3 ^d^	82.6 ^d^	80.0 ^d^	60.0 ^g^	47.9 ^f^	7.6 ^a^	53.7 ^f^	51.9 ^g^	47.9 ^f^	88.3 ^d^
fermented + enzymatic treated	84.8 ^c^	57.9 ^b^	44.0 ^a^	40.9 ^c^	14.8 ^b^	13.2 ^b^	20.0 ^c^	33.3 ^b^	32.4 ^c^	75.9 ^b^
germinated + fermented	93.7 ^e^	94.4 ^e^	61.0 ^c^	34.7 ^a^	24.7 ^c^	8.0 ^a^	20.4 ^c^	30.1 ^a^	22.8 ^a^	81.6 ^c^
enzymatic treated + fermented	85.4 ^c^	43.9 ^a^	44.9 ^a^	38.9 ^b^	8.3 ^a^	13.8 ^b^	18.6 ^b^	35.7 ^c^	33.7 ^c^	65.4 ^a^

Results were expressed as the mean of three replicates. Different letters within the same column (phenolic acids) indicate significant differences (*p* < 0.05; Duncan’s multiple range test).

## Data Availability

All data are contained in this article.
